# The HTRA1–Extracellular Matrix Axis in Health and Disease: From Pathological Remodeling to Therapeutic Opportunities in Fibrosis, Osteoarticular Disorders, and Cancer

**DOI:** 10.3390/cimb48100991

**Published:** 2026-09-25

**Authors:** Le Zong, Luyang Xiong, Yurou Wang, Manli Zhu, Jie Li, Jiayi Xu, Hunian Li, Li Yan

**Affiliations:** 1 Department of Critical Care Medicine, Tongji Hospital, Tongji Medical College, Huazhong University of Science and Technology, Wuhan 430030, China; zledoctor@163.com (L.Z.); dr.xiong@tjh.tjmu.edu.cn (L.X.); wyr980411@163.com (Y.W.); zml21673269817@outlook.com (M.Z.); lijie2022hust@163.com (J.L.); xujiayi_aa@163.com (J.X.); d202382272@hust.edu.cn (H.L.); 2 Department of Emergency, Tongji Hospital, Tongji Medical College, Huazhong University of Science and Technology, Wuhan 430030, China

**Keywords:** HTRA1, ECM regulation, fibrosis, osteoarticular diseases, cancer

## Abstract

High-temperature requirement A serine peptidase 1 (HTRA1) is a secreted serine protease that regulates extracellular matrix (ECM) homeostasis through direct proteolysis and indirect effects on matrix production and turnover. This review integrates evidence from fibrosis, bone and joint diseases, and cancer to examine how HTRA1 contributes to matrix remodeling and disease-associated cellular responses. In fibrosis, HTRA1 influences matrix accumulation by regulating collagen trafficking and secretion and transforming growth factor-β (TGF-β) signaling. In bone and joint diseases, HTRA1-mediated degradation of matrix components and induction of downstream proteases jointly contribute to tissue degeneration. In cancer, HTRA1 participates in tumor–stroma interactions through extracellular and intracellular mechanisms. Cross-disease comparison highlights the interplay between extracellular proteolysis, matrix production, and cellular signaling, alongside the tissue-dependent protective and pathogenic roles of HTRA1. We also summarize preclinical strategies for modulating HTRA1 and identify key gaps in current research. This review provides a conceptual framework for future mechanism-driven and translation-oriented research on HTRA1 in ECM-related diseases.

## 1. Introduction

### 1.1. Background

High-temperature requirement A serine peptidase 1 (HTRA1) is a member of the evolutionarily conserved HTRA family of serine proteases. This family of proteins is ubiquitously present in both prokaryotic and eukaryotic organisms, with initial identification in Escherichia coli as serine proteases induced by heat shock [1,2]. A defining feature of these proteins is their dual functionality, which includes both protease and molecular chaperone activity, thereby facilitating the regulation of protein homeostasis. Four human homologs have been identified: HTRA1, HTRA2, HTRA3, and HTRA4 [3,4]. These homologs collectively constitute a regulatory network that plays a crucial role in essential cellular processes.

HTRA family proteins exhibit significant structural homology, generally comprising at least one Protease Domain (PD) and one C-terminal PDZ domain [5]. Compared with other members of the HTRA family, HTRA1 has two unique structural characteristics in its domain composition. Its N-terminal sequence includes a Kazal-type protease inhibitory motif that modulates protease activity through an autoinhibitory mechanism. Unlike conventional HTRA family members, such as HtrA2, which depends on the PDZ domain for allosteric activation, the catalytic activity of HTRA1 operates independently of the PDZ domain. In HTRA1, the PDZ domain is solely involved in substrate binding and stabilization of oligomerization without contributing to the core activation function. These structural peculiarities may explain the functional disparities in HTRA1 and its divergence in associated pathophysiological processes compared with other HTRA family members (Table 1) [4,6].

As the first member of the HTRA family has been confirmed to exhibit secretory properties, HTRA1’s functional localization sets it apart from other family members. HTRA2 is predominantly localized in the mitochondria, where it plays a central role in the regulation of apoptosis [10]. Early studies on HTRA3 focused on pregnancy and female reproductive system diseases [6], whereas the expression of HTRA4 is tissue-restricted. Some studies have shown that the expression of human HTRA4 is limited to the placenta [13]. In contrast, HTRA1 can be secreted into the extracellular matrix (ECM), which directly influences regulation of the extracellular microenvironment. This distinctive expression and localization pattern positions HTRA1 as a crucial molecule that bridges intracellular signaling with the maintenance of extracellular microenvironment homeostasis [6].

### 1.2. ECM: A Dynamic Network in Health and Disease

The ECM constitutes a non-cellular component of tissue, characterized by a complex and highly dynamic three-dimensional structure that is secreted by cells into the extracellular space, providing essential structural and biochemical support. The ECM is primarily composed of various fibrous and non-fibrous network-forming macromolecules, including collagens, fibronectin, elastin, laminins, and nonstructural ECM proteins, such as enzymes, growth factors, and hormones, that play a crucial role in regulating transcellular signaling [17,18].

In addition to its biochemical functions, ECM provides physical cues that regulate cell behavior. Alterations in the ECM can affect tissue stiffness and force transmission. Cells sense and respond to these changes through mechanotransduction pathways involving integrins, focal adhesion-associated proteins, mechanosensitive ion channels, and cytoskeleton-coupled transcriptional regulators. The resulting signals can modify cellular contraction, differentiation, survival and matrix production [19,20].

Normal ECM function relies on a dynamic balance among matrix production, secretion, assembly, modification, degradation and clearance [21]. Matrix accumulation may result from increased synthesis or secretion, or insufficient degradation and clearance [22]. Conversely, tissue structural damage can arise from excessive proteolysis, inadequate replenishment after degradation, or impaired assembly of newly synthesized matrix. In addition, even when the total ECM content shows no obvious change, alterations in its composition, spatial organization or degree of crosslinking can still alter tissue stiffness and mechanical signaling [19]. Assessing ECM dysregulation therefore requires consideration of matrix composition, organization, turnover and mechanical properties, as well as matrix abundance.

HTRA1 can influence ECM homeostasis through mechanistically distinct pathways. First, extracellular HTRA1, a secreted serine protease, directly cleaves specific structural or matrix-associated substrates, including fibronectin, aggrecan, decorin, and collagen [23,24,25]. Second, HTRA1 indirectly modulates matrix production or degradation by regulating the transforming growth factor-β (TGF-β) pathway components [26,27,28,29,30,31], collagen trafficking and secretion [32], and downstream matrix-degrading proteases such as matrix metalloproteinases (MMPs) and A Disintegrin and Metalloproteinase with Thrombospondin motifs (ADAMTS) [33,34,35].

Building on this understanding, the “HTRA1–ECM axis” describes how HTRA1 influences cell behavior and disease progression through matrix remodeling (Figure 1). These actions can alter ECM abundance, composition, structure, and mechanical properties. These changes may, in turn, alter the biochemical and mechanical signals sensed by cells and modulate cellular functions, including adhesion and migration.

These matrix changes provide a common basis for understanding the roles of HTRA1 across diseases, although their biological effects vary with tissue and disease context. This review therefore uses ECM homeostasis as a framework to compare the roles of HTRA1 in fibrosis, bone and joint diseases, and cancer. We examine shared mechanisms and tissue-specific effects and discuss the potential of HTRA1 as a therapeutic target.

## 2. HTRA1 in Fibrotic Diseases

Fibrosis is characterized by the accumulation of ECM components within a specific tissue. As fibrosis advances, alterations in tissue composition occur, notably the accumulation of collagen and other ECM molecules. Excessive collagen observed during fibrosis primarily originates from interstitial fibroblasts, local mesenchymal stem cells (MSCs), endothelial cells, and damaged epithelial cells [36], with fibroblasts playing a pivotal role. Compared with their quiescent counterparts, activated myofibroblasts display increased ECM-synthetic activity. In many interstitial fibrotic lesions, the deposited matrix is enriched in fibrillar collagens, particularly types I and III, together with fibronectin and other matrix proteins [37]. Previous research has demonstrated that increased ECM deposition underlies nearly all cardiac diseases, underscoring the significant role of fibroblasts in fibrotic diseases [38].

Activated myofibroblasts deposit and reorganize fibrillar ECM while increasing matrix tension. The resulting stiff matrix further promotes cell spreading and actomyosin contraction and helps sustain the myofibroblast phenotype [20]. Integrin-dependent force transmission and cytoskeletal remodeling can translate matrix stiffness into persistent transcriptional and epigenetic responses, allowing fibroblasts to remain activated after the initial stimulus subsides [39]. Matrix stiffness may also act synergistically with soluble profibrotic signals. For instance, in skin fibrosis, high-stiffness matrices activate fibroblasts via the Piezo1-Wnt2/Wnt11-CCL24 pathway, and the resultant profibrotic responses further elevate tissue stiffness, forming a self-reinforcing loop [40]. These findings indicate that the organization and mechanical properties of the accumulated matrix can contribute to the persistence of fibroblast activation.

The profibrotic feedback between fibroblasts and the ECM is mediated by both mechanical and biochemical signals. Upon release into the ECM as active TGF-β, it interacts with its specific receptor (TGF-βR) on fibroblasts, thereby initiating downstream expression of profibrotic genes and resulting in fibroblast activation [41,42]. TGF-β further mediates a positive feedback loop for fibrosis between cells and ECM, wherein the feedback loop serves as a key mechanism through which HTRA1 regulates fibrotic diseases.

Keloid is a benign dermatological tumor characterized by excessive proliferation of fibroblasts and ECM deposition, often resulting in suboptimal treatment outcomes and a high rate of recurrence [43]. HTRA1 is highly expressed in human keloid skin tissues [23]. Silencing the HTRA1 gene has been shown to effectively inhibit the proliferation of Keloid Fibroblasts (KFBs) and reduce ECM deposition [44]. Jin et al. demonstrated that HTRA1 is implicated in the regulation of latent TGF-β1 activation in KFBs, thereby leading to increased Smad2 phosphorylation, which may represent one of the mechanisms underlying keloid formation [23]. Further research by Wang et al. confirmed that microRNA-384 (miR-384) can target and negatively regulate HTRA1 expression. However, miR-384 is downregulated in keloids, which promotes KFB proliferation and suppresses apoptosis due to increased inhibition of HTRA1 expression [45]. Consequently, HTRA1 may represent a potential therapeutic target for keloid treatment in the future.

Recent advancements have improved our understanding of the role of HTRA1 in fibrosis-related diseases affecting parenchymal organs. Shi et al. found that HTRA1 expression is upregulated in patients with dilated cardiomyopathy (DCM) and is closely correlated with myocardial fibrosis. The underlying mechanism involves HTRA1’s recruitment of SEC16A, which enhances the formation of endoplasmic reticulum exit sites (ERES). This process facilitates the transport of collagen type I (Col1) from the endoplasmic reticulum to the Golgi apparatus, thereby promoting efficient Col1 secretion [32]. These findings indicate that HTRA1 can contribute to ECM remodeling by regulating the trafficking and secretion of newly synthesized collagen. Quinn et al. demonstrated that the expression of soluble folate receptor γ (FOLR3) is upregulated in the livers of patients with myeloproliferative neoplasm-associated liver disease (MASH), and that it interacts with HTRA1. Previous studies have demonstrated that HtrA1 negatively regulates the TGF-β signaling pathway by degrading transforming growth factor beta receptor II (TGFBR2) [26]. However, binding of FOLR3 to HTRA1 inhibits this degradation, thereby augmenting TGF-β signaling. This enhancement promotes the activation of stellate cells, increases the production of ECM proteins, and ultimately exacerbates liver fibrosis [46].

Collectively, the fibrotic mechanisms discussed in this section mainly influence ECM deposition through changes in signaling and collagen secretion. By regulating latent TGF-β1 activation, receptor stability, and collagen secretory trafficking, HTRA1 can alter profibrotic signaling or the delivery of newly synthesized collagen to the extracellular space. Although cleavage of TGFBR2 is a direct proteolytic action of HTRA1, its effect on ECM deposition is indirect, occurring through changes in cellular signaling and matrix production. Therefore, the net effect of HTRA1 on fibrosis depends on multiple factors, including its molecular targets and subcellular localization.

## 3. HTRA1 in Joint and Skeletal Disorders

Considering the pivotal roles of HTRA1 in cellular differentiation [25,47,48,49,50] and ECM remodeling [33,51,52], extensive studies have been conducted to elucidate its function in skeletal diseases (SDs). Both osteoblasts and osteoclasts produce and secrete HTRA1 [25,50]. By regulating TGF-β and bone morphogenetic protein (BMP) pathways [23] and degradation of BMP2, BMP4, and TGF-β1, HTRA1 fine-tunes bone formation [53].

The role of HTRA1 in osteoporosis is not yet fully understood and there are varying research perspectives. Evidence indicates that HTRA1 expression is upregulated during the differentiation of MSCs into osteoblasts, and that recombinant HTRA1 enhances the osteogenic differentiation of human bone marrow MSCs, suggesting a positive influence on bone formation [48]. Conversely, HTRA1 inhibits osteoblast differentiation and promotes osteoclastogenesis through the degradation of osteoprotegerin (OPG), thereby disrupting bone remodeling homeostasis [47,50]. HtrA1 influences bone metabolism in a bidirectional manner that is dependent on cellular sources. Specifically, when secreted by cells of osteoblastic lineage, HtrA1 primarily facilitates bone formation and matrix maturation [54]. Conversely, when secreted by osteoclasts, it promotes bone resorption through degradation of osteoprotegerin (OPG) [50]. These dual roles collectively contribute to the regulation of bone remodeling. Furthermore, HTRA1 modulates angiogenesis by interacting with the TGF-β pathway, indirectly affecting bone metabolism [53,55].

HTRA1 is also involved in rheumatoid arthritis (RA). Studies have shown that HTRA1 can directly degrade ECM components. Recombinant HTRA1 cleaves fibronectin into multiple products, including a prominent 29–30 kDa fragment. Unlike intact fibronectin, HTRA1-digested fibronectin induces the expression of MMP1 and MMP3 in human synovial fibroblasts [35]. These findings support the presence of bioactive components within HTRA1-generated fibronectin fragment mixtures that can stimulate cellular responses. Nevertheless, because the active constituent in this mixture of fragments has not been individually isolated, its precise molecular identity and cellular receptor remain undefined. The expression of HTRA1 is modulated by various cytokines, such as lipopolysaccharide (LPS), which markedly upregulates HTRA1 expression through activation of the Toll-like receptor 4 (TLR4)/NF-κB signaling pathway, whereas interferon-γ (IFN-γ) suppresses HTRA1 expression via the p38 MAPK/STAT1 pathway. The administration of anti-HTRA1 neutralizing antibodies has been shown to significantly ameliorate disease symptoms and severity in a collagen-induced arthritis mouse model, indicating that HTRA1 may represent a viable therapeutic target for RA [56,57]. Some active compounds derived from plant extracts, such as Polygonum orientale L., can mitigate RA symptoms, which are directly attributable to the formation of stable complexes with HTRA1 [58].

Osteoarthritis (OA) is a degenerative joint disorder closely associated with HTRA1 expression. In the cartilage tissue of OA patients, HTRA1 mRNA levels are markedly elevated, correlating with disease progression [59,60]. HTRA1 can degrade various components of cartilage, including aggrecan, fibromodulin, decorin, and soluble type II collagen, thereby inducing cartilage degeneration [61]. Studies have also revealed that the TGF-β1 pathway induces the expression of HTRA1 in OA, and that the interaction between TGF-β1 and HTRA1 contributes to the degradation of articular cartilage [62,63]. Therapeutic investigations targeting HTRA1 have led to several notable advancements. Compounds, such as Wogonin and Losartan, have been shown to reduce HTRA1 expression and mitigate inflammatory responses and OA progression [64,65]. Furthermore, marine-derived collagen hydrolysates, such as Promerim, have also been found to downregulate the expression of HTRA1 and other related factors, promote chondrocyte anabolism, and thus possess potential therapeutic value for the treatment of OA [66,67].

Intervertebral disc degeneration (IVDD) is a common cause of chronic low back pain. Published evidence shows a positive correlation between HTRA1 expression and IVDD severity, suggesting that HTRA1 facilitates IVDD progression through multiple mechanisms [34,52,68,69]. Studies in intervertebral disc degeneration provide further evidence linking HTRA1-mediated fibronectin cleavage to downstream MMP induction. Proteolytically active HTRA1 increases the expression of MMP1, MMP3 and MMP13 as well as MMP3 secretion in human IVD cells, whereas catalytically inactive HTRA1 mutants exert markedly weaker effects. HTRA1 generates a fibronectin fragment containing the N-terminal fibrin- and heparin-binding domains. After removal of most residual recombinant HTRA1, HTRA1-digested fibronectin preparations still induce the expression of MMP1, MMP3 and MMP13. Meanwhile, MEK inhibition also attenuates HTRA1-induced MMP1 and MMP3 expression, supporting a role for MEK signaling in this response [34]. Additionally, HTRA1 modulates signaling pathways, including ERK, NF-κB, and JNK, to enhance the expression of proteins, such as ADAMTS-5, further exacerbating IVDD [33]. Targeting HTRA1 or its associated signaling pathways may represent a promising therapeutic approach for the management of IVDD.

Collectively, evidence from osteoarticular diseases supports two mechanistically distinct but potentially connected routes of matrix degradation. The first is direct extracellular proteolysis, in which HTRA1 cleaves structural ECM substrates, including aggrecan, fibromodulin, decorin, type II collagen, and fibronectin. The second involves indirect regulation of downstream matrix-degrading proteases. Responses to HTRA1-generated cleavage products provide one potential link between these routes. Mixed preparations containing HTRA1-generated fibronectin fragments can stimulate MMP production in synovial fibroblasts and intervertebral disc-resident cells. The resulting increase in MMP production could amplify matrix degradation beyond the initial cleavage events mediated by HTRA1. These findings support a plausible feedforward amplification mechanism.

However, the studies discussed here used mixtures of fibronectin fragments and did not establish which individual fragment was necessary and sufficient for cellular activation. Identifying the active fragments and their receptors, and establishing the sequence of HTRA1 and MMP activities, would help determine whether this proposed amplification contributes to sustained tissue degeneration.

## 4. Context-Dependent Regulation of TGF-β Signaling by HTRA1

The differing effects of HTRA1 on TGF-β signaling involve distinct substrates and their roles within the pathway (Figure 2). In studies of cerebral small vessel disease, secreted HTRA1 cleaves LTBP1, weakening its interaction with fibronectin and its matrix anchorage, thereby favoring subsequent TGF-β signaling activation [27]. HTRA1 has also been observed to promote TGF-β signaling activation in keloids [23]. By contrast, direct cleavage of TGF-β1 [70] or cell-surface TGFBR2 and TGFBR3 [26] inhibits signaling by reducing ligand or receptor availability. These findings indicate that the effect of proteolysis on the pathway depends on the function of the cleaved molecule. Altering the matrix anchorage of latent complexes and reducing the availability of ligands or receptors can have opposing effects on signaling.

The extracellular or intracellular localization of HTRA1 further distinguishes the steps it regulates. Extracellular HTRA1 can act on matrix-associated proteins, ligands, and cell-surface receptors. Intracellular HTRA1 can cleave the prodomain of pro-TGF-β1 in the endoplasmic reticulum, contributing to precursor processing [71]. TGF-β1 can also induce HTRA1 expression in chondrocytes [72], indicating that regulation occurs in the reverse direction as well.

HTRA1 thus acts as both a regulator of and a responder to TGF-β signaling. Its actions at different steps contribute to the complex regulatory relationship between HTRA1 and this pathway.

## 5. HTRA1 in Cancer Development and Progression

Although HTRA1 has been implicated in the development of numerous cancers, including those affecting the breast, endometrium, ovary, liver, lung, esophagus, and bladder [73,74,75], its role is multifaceted and complex.

### 5.1. Oncogenesis and Early Diagnosis of Cancer

In colorectal cancer, there is an inverse correlation between HTRA1 expression and UC duration of ulcerative colitis. Specifically, the level of HTRA1 in colonic tissues of patients with ulcerative colitis persisting for over 10 years was markedly diminished, paralleling the reduction observed in cancerous tissues of individuals with colorectal cancer [76]. Similarly, in urothelial bladder cancer, reduced HTRA1 levels were detected in both cancerous tissues and the urine of affected patients [74]. In esophageal cancer tissues, the expression of HTRA1, both at the mRNA and protein levels, was reduced compared with that in the adjacent normal tissues. A similar reduction was observed in gastric cancer tissues [77,78]. HTRA1 directly suppressed the proliferation of pancreatic cancer cells [79].

Current evidence concerning HTRA1 in early tumorigenesis comes mainly from expression changes in patient tissues or body fluids and functional studies in cancer cells. Whether these findings involve ECM remodeling remains unresolved in most settings. We briefly summarize them as background to the broader cancer-related functions and potential biomarker relevance of HTRA1, while distinguishing them from evidence supporting the HTRA1–ECM axis.

### 5.2. Cancer Metastasis and Epithelial–Mesenchymal Transition (EMT)

Reduced HTRA1 expression is associated with metastatic progression in multiple cancers. In breast cancer, HTRA1 loss correlates with enhanced proliferative activity and metastatic behavior, and the absence of HTRA1 in patient sentinel lymph nodes is also linked to an increased probability of non-sentinel lymph node metastasis [80,81]. In endometrial carcinoma, reduced HTRA1 expression is similarly associated with enhanced invasive capacity and higher tumor grade [82,83]. Reduced HTRA1 expression correlates with lymph node metastasis and poor prognosis in esophageal cancer [78]. Studies have reported that HTRA1 levels are lower in metastatic sites than in primary sites in ovarian and gastric cancers [84,85], suggesting a strong association between HTRA1 downregulation and cancer metastasis.

Several studies further link this association to pathways governing cell migration and epithelial–mesenchymal plasticity. In breast cancer, the interaction between HTRA1 and proteoglycan link protein 1 modulates EGF/EGFR signaling, thereby affecting cell migration and stemness-like traits [86]. Moreover, reduced HTRA1 expression also predicts poorer survival in the metastatic setting [80,87]. In hepatocellular carcinoma (HCC), reduced HTRA1 expression is associated with lower E-cadherin and higher vimentin expression in human tissues and cell lines [88].

Although these studies link HTRA1 to invasion, migration and EMT-associated phenotypes, the contribution of ECM remodeling and mechanical signaling to these effects remains unclear. Given its involvement in ECM proteolysis and remodeling, HTRA1 may influence tumor cell invasion beyond its direct effects on intracellular signaling. It may also indirectly shape invasive behavior by altering the surrounding matrix environment. ECM composition, organization, and mechanical properties can influence interactions between tumor cells and their microenvironment. For example, increased collagen deposition and crosslinking can stiffen the matrix and activate signaling programs such as YAP/TAZ through mechanotransduction involving integrins, focal adhesions, and cytoskeletal tension. These signals can, in turn, affect cell migration, survival, and phenotypic plasticity [17,19,89]. A stiff matrix can also sustain CAF activation, supporting continued ECM deposition and reorganization by these cells and creating a positive feedback loop between ECM stiffness and CAF activation [90,91].

ECM remodeling may therefore provide an important intermediate link between HTRA1 dysregulation and invasive tumor phenotypes. HTRA1 has been reported to participate in the conversion of fibroblasts into CAFs in gastric cancer. HTRA1 overexpression in gastric cancer cells can enhance NF-κB p65 phosphorylation and FGF2 secretion, inducing the upregulation of α-SMA and other molecules in normal fibroblasts and promoting CAF-associated phenotypes [92]. This mechanism supports an indirect effect of HTRA1 on the tumor stroma and suggests that HTRA1 may act upstream of CAF-mediated ECM remodeling. Fibroblasts that acquire CAF-associated phenotypes may, in turn, contribute to further ECM changes.

A recent study in colorectal cancer identified an intracellular mechanism linking HTRA1 to YAP1 regulation. HTRA1 binds to and stabilizes LATS2, thereby maintaining YAP1 phosphorylation and limiting its nuclear accumulation. HTRA1 loss promotes LATS2 degradation, YAP1 activation, EMT-associated changes, and resistance to anoikis [73]. Because YAP1 is also a mechanosensitive transcriptional regulator, the HTRA1-LATS2-YAP1 pathway may represent a point of convergence between intracellular HTRA1 signaling and ECM-dependent mechanotransduction [89]. Future studies should examine this pathway in experimental systems with defined mechanical conditions to determine whether it transmits ECM-derived signals or acts independently of the ECM.

Together, these findings suggest context-dependent links between HTRA1, tumor cell signaling and stromal remodeling, although the contribution of ECM mechanics remains to be established.

### 5.3. HTRA1 in Cancer Therapy Response: Mechanisms Without an Established ECM Link

Beyond its ECM-associated functions, HTRA1 can also modulate responses to cancer therapy through intracellular signaling and alterations in protein abundance. The most extensively studied mechanism involves the X-linked inhibitor of apoptosis protein (XIAP). In liver and ovarian cancer models, HTRA1-mediated XIAP degradation promotes chemotherapy-induced apoptosis and enhances cisplatin sensitivity [93,94]. In colorectal cancer, HTRA1 downregulation leads to XIAP accumulation and activation of PI3K/AKT signaling, which in turn facilitates cisplatin resistance [95]. Collectively, these findings support the HTRA1-XIAP axis as a core regulator of chemoresistance across multiple cancer types.

In gastric cancer, high HTRA1 expression correlates with favorable responses to cisplatin-containing combination chemotherapy and longer overall survival [96]. In non-small cell lung carcinoma, reduced HTRA1 expression mediated by the HDAC/RXR regulatory axis promotes cisplatin resistance, whereas restoration of HTRA1 expression resensitizes cancer cells to cisplatin [97], providing experimental rationale for further investigating whether HTRA1 re-expression can overcome cisplatin resistance.

Although HTRA1 is frequently described as a tumor suppressor, available evidence does not support a consistent function across all cancers (Table 2). In ovarian and colon cancer cells, HTRA1 enhances chemotherapy-induced apoptosis via XIAP-related pathways. In colorectal cancer models, HTRA1 has been linked to increased cisplatin sensitivity through XIAP-related signaling, whereas its interaction with SLC7A11 has been associated with resistance to 5-FU and oxaliplatin [93,95,98]. Similar discrepancies exist regarding tumor progression. In ovarian, gastric and breast cancers, loss or reduced expression of HTRA1 is associated with increased invasion, anoikis resistance, or poor survival [77,84,85,87]. Conversely, elevated HTRA1 has been implicated in KLF13-driven breast cancer progression and gastric cancer cell-mediated conversion of normal fibroblasts into CAFs [92,99]. These findings show that HTRA1 expression alone does not consistently predict its biological effects. Differences in cellular source, localization and molecular interactions may contribute to the divergent outcomes, although their relative contributions remain unresolved.

**Table 2:** Functions of HTRA1 in cancer: mechanistic and observational evidence. (**A**) Mechanistic and functional evidence. (**B**) Clinical and bioinformatic association evidence.

(**A**)					
**Cancer Type**	**Cellular Source and Reported Localization of HTRA1**	**HTRA1-Associated Molecular Mechanism**	**Functional Role of HTRA1**	**Biological Outcome**	**References**
Breast cancer	Breast cancer cells	KLF13 regulates HTRA1 transcription; HTRA1 is implicated in Hedgehog/GLI1 and cancer stemness-related signaling, accompanied by altered CD8^+^ T-cell activity	Tumor-promoting	Restoring HTRA1 expression reverses the reductions in cell proliferation, migration, mammosphere formation, and xenograft growth caused by KLF13 knockdown	[99]
Gastric cancer	Gastric adenocarcinoma cells	Not reported	Tumor-suppressive	HTRA1 overexpression suppresses cell proliferation, migration, and Matrigel invasion	[85]
Gastric cancer	Gastric cancer cells	HTRA1 overexpression enhances NF-κB p65 phosphorylation and bFGF secretion, thereby inducing a CAF-like phenotype in fibroblasts	Promotes a CAF-like phenotype	Conditioned medium induces α-SMA and other CAF-associated markers in normal fibroblasts	[92]
Ovarian cancer	Ovarian cancer cells and primary ovarian tumors	HTRA1 induces cell death through a protease activity-dependent mechanism	Tumor-suppressive	HTRA1 loss promotes anchorage-independent growth, whereas restoration of proteolytically active HTRA1 induces cell death	[100]
Ovarian cancer	Ovarian cancer cells; HTRA1 is predominantly	HTRA1 promotes anoikis in a protease activity-dependent manner and suppresses EGFR-AKT survival signaling	Tumor-suppressive and anti-dissemination	HTRA1 loss promotes anoikis resistance and peritoneal dissemination	[84]
Ovarian cancer	Ovarian cancer cells	HTRA1 directly cleaves and degrades XIAP, leading to caspase-3/7 activation; this effect requires HTRA1 protease activity	Chemosensitizing	HTRA1 enhances cisplatin-induced apoptosis	[93]
Colon cancer	Colon adenocarcinoma cells	HTRA1 downregulation leads to XIAP accumulation and PI3K-AKT activation	Chemosensitizing	HTRA1 loss promotes cisplatin resistance, whereas restoring HTRA1 expression increases cisplatin sensitivity	[95]
Colorectal cancer	Colorectal cancer cells; HTRA1 is predominantly cytoplasmic	HTRA1 binds and stabilizes SLC7A11 through its Kazal domain, sustaining the SLC7A11-GPX4-GSH antioxidant axis and thereby suppressing lipid peroxidation and ferroptosis	Tumor-promoting and chemoresistance-promoting	By suppressing ferroptosis, HTRA1 promotes tumor growth and resistance to 5-FU and oxaliplatin	[98]
(**B**)					
**Cancer Type**	**Study Design**	**Main Observation**		**Interpretation Supported by the Evidence**	**References**
Breast cancer	Clinical expression–survival analysis of bulk tumor tissue	Low HTRA1 expression is associated with disease progression and poorer survival		Supports the prognostic relevance of HTRA1	[87]
Breast cancer	Predominantly bulk-tumor transcriptomic and bioinformatic analyses	Low HTRA1 expression is associated with poorer survival and multiple immune-cell- and proliferation-related transcriptional signatures		Supports a hypothesis linking HTRA1 to the tumor immune microenvironment	[101]
Gastric cancer	Expression analysis of tumor tissues and gastric cancer cell lines	HTRA1 expression is reduced in gastric cancer, and its downregulation is associated with promoter methylation		Establishes an association between altered HTRA1 expression and epigenetic regulation	[77]
Gastric cancer	Clinical IHC and treatment-outcome association analysis	High HTRA1 expression is associated with a better chemotherapy response and longer overall survival		Supports HTRA1 as a potential predictive biomarker	[96]
High-grade serous ovarian carcinoma	Compartment-specific IHC assessment of nuclear and cytoplasmic HTRA1 in tumor cells	Low nuclear HTRA1 expression is associated with lower risks of disease progression and death, whereas cytoplasmic HTRA1 has no prognostic value		Supports a localization-specific association with prognosis	[102]

Abbreviations: CAF, cancer-associated fibroblast; EGFR, epidermal growth factor receptor; 5-FU, 5-fluorouracil; GSH, glutathione; IHC, immunohistochemistry; XIAP, X-linked inhibitor of apoptosis protein.

These opposing outcomes may be explained by several mechanistic differences. Protease-dependent cleavage of intracellular substrates, exemplified by XIAP degradation, favors apoptosis and chemosensitivity [93]. HTRA1 interacts with SLC7A11 via its Kazal domain to inhibit ferroptosis, and this effect has not been shown to rely on HTRA1 protease activity [98]. The cellular recipient of HTRA1-associated signals may also influence the observed outcome. In gastric cancer, tumor cell-derived HTRA1 promotes NF-κB-dependent bFGF secretion, which subsequently induces CAF transformation. Yet another study in the same cancer type reported that HTRA1 suppresses proliferation, migration and invasion of gastric cancer cells themselves [85,92]. In high-grade serous ovarian carcinoma, low nuclear HTRA1, but not cytoplasmic HTRA1, is associated with better clinical outcome [102]. These observations suggest that localization warrants further investigation but do not establish its functional contribution.

Table 2 separates findings from functional and mechanistic experiments from clinical and bioinformatic associations. Although these studies identify distinct HTRA1-associated mechanisms, they do not establish a unified explanation for the opposing outcomes. Further studies are needed to determine how cellular source, localization and specific molecular interactions contribute to these differences.

### 5.4. Therapeutic Prospects and Challenges of Targeting HTRA1

The effects of HTRA1 on tumor progression and treatment response have prompted efforts to modulate its expression or activity. Table 3 groups HTRA1-related interventions into three categories: direct or putatively direct targeting strategies, drugs that indirectly alter HTRA1 expression or protein abundance, and experimental genetic or biochemical interventions. Strategies targeting pathways upstream of HTRA1 are included in the second category.

Direct intervention strategies include carfilzomib, a candidate HTRA1 inhibitor identified through virtual screening that suppresses malignant phenotypes in models of pancreatitis-associated pancreatic ductal adenocarcinoma (PDAC) [104]. However, its pharmacological specificity for HTRA1 remains uncertain, and it is therefore classified as a putatively direct targeting strategy. Experimental nucleic acid-based approaches have also been explored, including lipid nanoparticle delivery of anti-HTRA1 mRNA to downregulate HTRA1 expression [103].

Indirect interventions primarily alter HTRA1 expression or activity through upstream pathways. WT-161 inhibits HDAC6 and restores HTRA1 expression in colorectal cancer cells [106,107]. DW22 modulates the HDAC/RXR axis, restoring HTRA1 expression and cisplatin sensitivity in drug-resistant non-small-cell lung cancer cells. In a mouse model of peritoneal mesothelioma, piroxicam treatment increases HTRA1 expression and suppresses tumor growth [105]. However, these studies did not establish the direct pharmacological targets of the drugs; they identify HTRA1 as an important mediator of their effects.

However, targeting HTRA1 therapeutically still faces considerable challenges. First, HTRA1 is not a monolithic target; its functions vary substantially across tissues and cell types. In osteoarticular diseases, extracellular HTRA1 contributes to the pathological degradation of cartilage or intervertebral disc matrix, providing a rationale for inhibiting extracellular HTRA1 protease activity [56,58,64,65]. By contrast, in some cancers, intracellular HTRA1 promotes XIAP degradation and enhances apoptosis and chemotherapy responses [93,94]. Thus, in tumors that retain this protective function, broadly reducing HTRA1 expression or inhibiting its intracellular protease activity may be detrimental. Even within the skeletal system, the divergent roles of HTRA1 in osteoblast and osteoclast lineages suggest that therapeutic outcomes may depend on the cell population targeted and the balance between bone formation and resorption [47,48,50,54]. Systemic HTRA1 inhibition could therefore suppress pathological extracellular proteolysis while also impairing beneficial intracellular functions or altering bone remodeling in other tissues.

Second, protease-dependent functions should be distinguished from functions whose dependence on catalytic activity remains unresolved. Direct cleavage of ECM substrates, XIAP or TGFBR2 requires HTRA1 protease activity [26]. HTRA1 also interacts with SLC7A11 through its Kazal domain in colorectal cancer [98] and with SEC16A during collagen secretion in cardiac fibroblasts [32]. These interactions alone do not establish whether the resulting effects require HTRA1 catalytic activity. Where this remains unresolved, studies using catalytically inactive HTRA1 or selective activity inhibitors could help clarify whether catalytic inhibition would alter these functions.

Therapeutic development should therefore consider the disease context, cellular compartment and molecular function targeted.

## 6. Conclusions and Perspectives

Across the disease contexts covered in this review, HTRA1 does not exert a uniformly protective or pathogenic role. These differences imply that systemic inhibition may reduce pathogenic extracellular proteolysis in some disease contexts but may also suppress the intracellular HTRA1 functions that promote apoptosis or enhance chemosensitivity in certain cancers. Conversely, systemic restoration of HTRA1 may restore protective functions in one context yet enhance disease-promoting effects in another.

Spatially selective interventions can help reduce these risks. Possible approaches include local administration, tissue-targeted delivery, and cell-selective carriers. For example, inhibiting extracellular HTRA1 may offer therapeutic benefit where excessive pericellular matrix proteolysis has been established as a disease driver. For anatomically accessible lesions, local administration can reduce systemic exposure. In tissues less amenable to direct local intervention, tissue-targeted delivery can increase the accumulation of therapeutic molecules at disease sites, thereby limiting HTRA1 perturbation in non-target tissues. Carriers designed to exploit specific cell-surface receptors or microenvironmental features can further restrict interventions to disease-relevant cell populations. This can improve spatial precision and reduce interference with the physiological functions of HTRA1 in other cells.

However, tissue- or cell-level targeting alone cannot distinguish between the intracellular and extracellular functions of HTRA1. Selective inhibition of extracellular HTRA1 therefore requires additional control over where therapeutic molecules act, favoring activity in the extracellular space while minimizing cellular entry and effects on intracellular HTRA1 activity.

Functional selectivity is equally important. Where pathological effects depend on HTRA1-mediated proteolysis, selectively modulating catalytic activity may offer an alternative to changing total HTRA1 expression. The effects on other protease-dependent functions would still need to be assessed. A more selective strategy would target disease-driving interactions between HTRA1 and specific substrates or binding partners while preserving its other functions. Developing such interventions requires identifying causally relevant substrates or binding partners, delineating the corresponding interaction interfaces, and establishing biomarkers capable of distinguishing HTRA1 expression, localization, catalytic activity, and substrate-binding status. These approaches remain to be validated for the proposed applications, including their ability to preserve beneficial HTRA1 functions while limiting disease-promoting effects.

HTRA1 is a potential therapeutic target whose value depends on the underlying mechanism. Tissue-selective delivery, targeting more clearly defined cellular sources, and selective modulation of molecular interactions offer possible routes for translating its complex molecular mechanisms into precision therapies. These approaches may help narrow the gap between basic research and clinical application.

The regulation of ECM by HTRA1 in fibrotic diseases, bone and joint disorders, and cancer illustrates the diversity and complexity of its functions. HTRA1 may emerge as a key ECM regulator involved in additional ECM-related diseases, including renal fibrosis resulting from chronic kidney injury. These potential roles warrant further investigation.

## Figures and Tables

**Figure 1:**
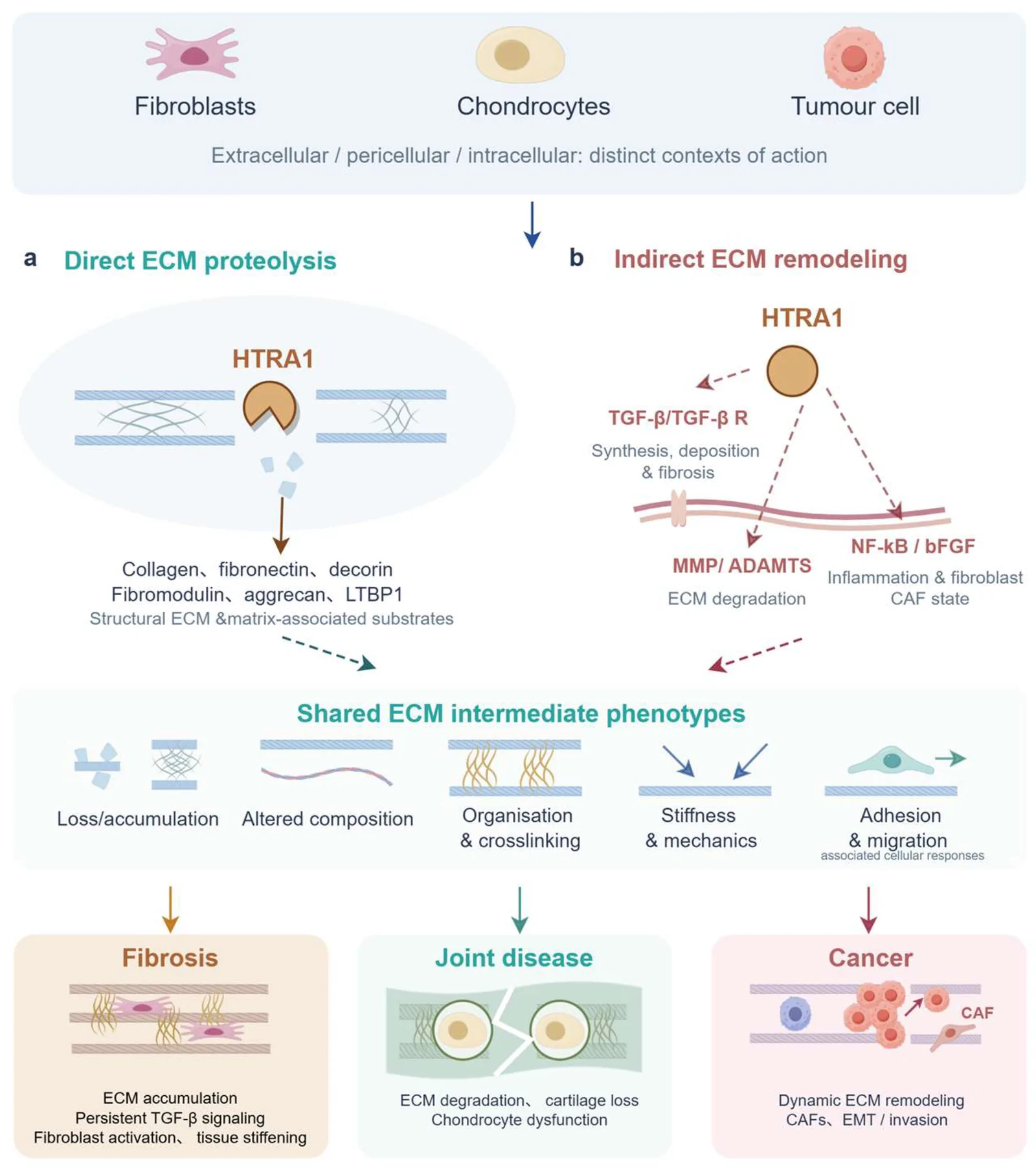
The HTRA1–ECM axis links context-dependent matrix remodeling to disease-specific outcomes. (**a**) HTRA1 proteolysis of structural ECM components and matrix-associated substrates, including collagen and fibronectin, can alter matrix architecture and growth factor storage. (**b**) HTRA1 can also indirectly influence ECM synthesis, deposition, and degradation, as well as fibroblast and cancer-associated fibroblast (CAF) states, through context-dependent mechanisms involving pathways such as TGF-β/TGF-βR signaling. Together, these effects can lead to excessive ECM degradation or abnormal deposition and changes in matrix composition, organization, and crosslinking. They can also alter matrix stiffness, mechanical signaling, and signals regulating cell adhesion and migration. However, the associated pathological outcomes vary across tissue contexts. Dashed lines indicate indirect or context-dependent relationships and do not imply a uniform direction of activation or a hierarchy of evidence strength.

**Figure 2:**
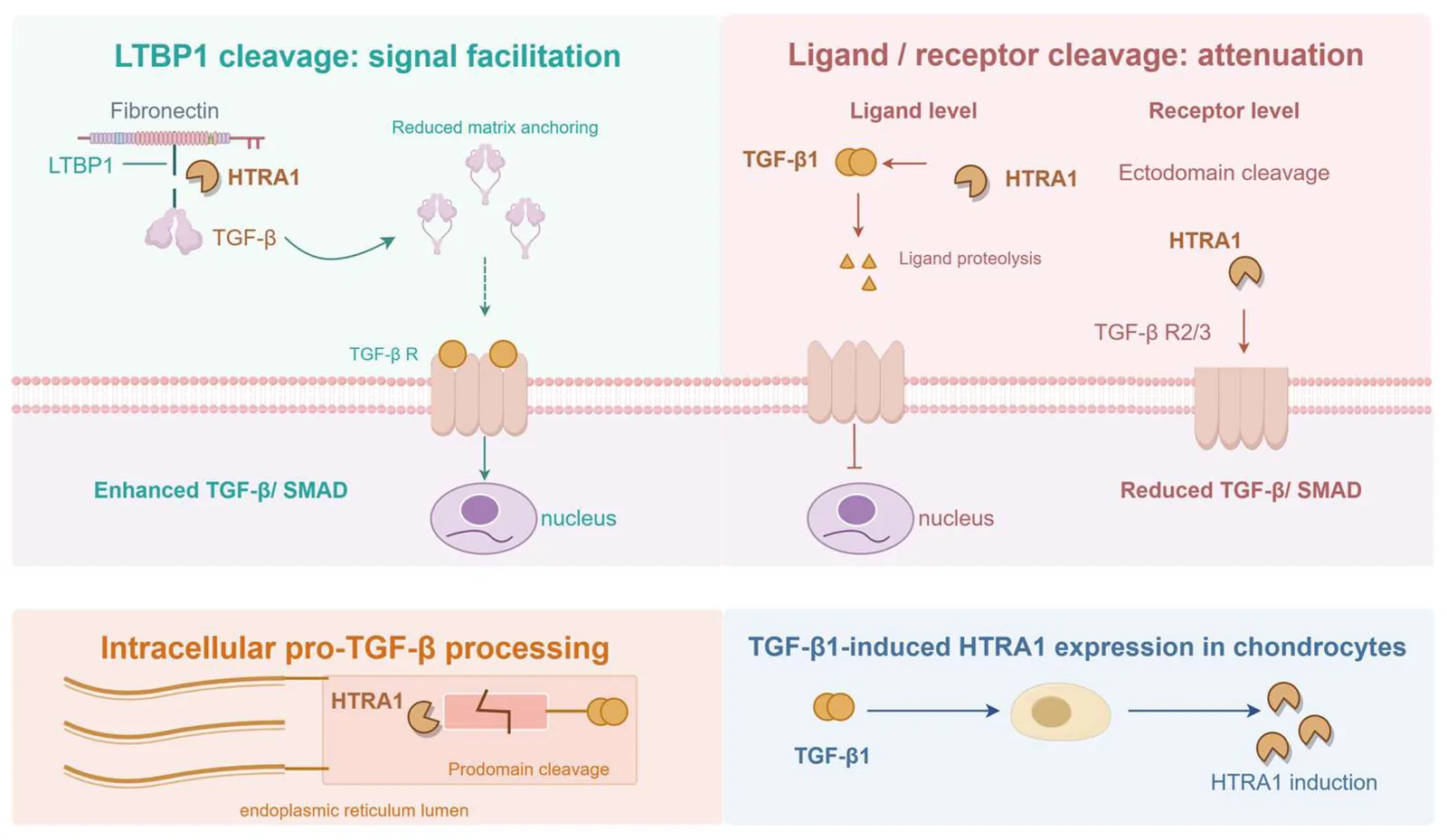
Mechanisms underlying the opposing effects of HTRA1 on TGF-β signaling. HTRA1 cleaves LTBP1, reducing its association with fibronectin and matrix anchorage and facilitating subsequent TGF-β activation. LTBP1 cleavage does not itself release mature, active TGF-β. The dashed arrow denotes an indirect link to downstream signaling rather than a single cleavage-mediated activation step.

**Table 1:** Comparison of Core Characteristics of HTRA Family Members.

Characteristics		HTRA1	HTRA2	HTRA3	HTRA4
Trimer		Structural unit, capable of higher-order oligomerization	Existing form, pyramid-like structure	Structural unit, PDZ domain affecting its formation	Main existing form (minor monomer), substrate binding stabilizing it
Core Domain	Shared Segment	One PD and one PDZ domain;	One PD and one PDZ domain	One PD and one PDZ domain	One PD and one PDZ domain
	N-terminus	Cleavable signal peptide and IGFBP7 fragment (contains one Kazal-type protease inhibitor motif)	Transmembrane anchor sequence (processable for removal)	Cleavable signal peptide and IGFBP7 fragment	Cleavable signal peptide and IGFBP7 fragment
Protein Type		Secretory pathway-targeted protein	Non-secretory pathway-targeted protein	secretory pathway-targeted protein	Presumably a non-secretory pathway-targeted protein
Cellular Localization		Mainly secreted to ECM; ~20% localized to cytoplasm (microtubule-bound) & plasma membrane	Mainly localized to mitochondrial intermembrane space (IMS); small amount present in nucleus; released to cytoplasm during apoptosis	ECM	Detailed localization not specified;
Core Function		Protein quality control; regulation of cell proliferation and migration; involvement in ECM remodeling	Mitochondrial quality control; degradation of abnormal proteins; regulation of apoptosis	Induction of mitochondria-mediated apoptosis; involvement in placenta development-related processes	Involvement in the apoptotic pathway
Associated Diseases		Osteoarticular system diseases; cancer; fibrotic diseases; AD; AMD	PD; AD; cancer	Endometrial cancer; ovarian cancer; lung cancer; PE	PE; GBM; breast cancer; prostate cancer
References		[4,6,7]	[8,9,10]	[7,11,12]	[13,14,15,16]

Abbreviations: PD, protease domain.

**Table 3:** Preclinical interventions and experimental manipulations associated with HTRA1 in cancer.

Evidence Category	Cancer Type	Intervention	Mechanism	Main Findings and Limitations	References
Direct HTRA1-targeting strategy	PDAC	LNP delivery of mRNAs encoding the heavy and light chains of an anti-HTRA1 antibody (alpha-HTRA1 mRNA)	Neutralizes HTRA1 and reduces HTRA1 protein abundance, thereby suppressing HIF-1-associated redox programs	Inhibited proliferation, migration, invasion, and organoid progression. Evidence from intact tumor-bearing animal models and clinical studies is lacking.	[103]
Putative direct HTRA1-targeting strategies	Pancreatitis-initiated PDAC	Carfilzomib identified by structure-based virtual screening	Inhibition of HTRA1/CDK1	Suppressed malignant phenotypes and PDAC progression. Carfilzomib is an established proteasome inhibitor, and its HTRA1-specific pharmacological activity remains unresolved.	[104]
	Multidrug-resistant hepatocellular carcinoma	NVP-LEB748, reported as an HTRA1 inhibitor, combined with HTRA1 overexpression experiments	HTRA1/XIAP/caspase-associated chemosensitivity	HTRA1 overexpression restored chemosensitivity, whereas the inhibitor reversed the associated molecular effects.	[94]
Agents that indirectly regulate HTRA1 expression or abundance	Peritoneal mesothelioma	Piroxicam alone or combined with cisplatin	Increased HTRA1 and decreased EGFR	Suppressed tumor growth and enhanced the cisplatin response; HTRA1 causality was not separated from the pleiotropic effects of the drug.	[105]
	Colon cancer	HDAC6 inhibitor WT-161	HDAC6 inhibition restored HTRA1 expression	Restored HTRA1 and attenuated malignant phenotypes; animal and clinical therapeutic validation is lacking.	[106]
	Cisplatin-resistant non-small-cell lung cancer	DW22 targeting HDAC/RXR	Modulated HDAC/RXR and restored HTRA1 expression and cisplatin sensitivity	Supports pharmacological modulation of an HTRA1-associated regulatory axis but does not establish DW22 as a direct HTRA1-targeting agent.	[97]
Experimental genetic or biochemical manipulation of HTRA1	Esophageal squamous cell carcinoma	HTRA1 overexpression	Reduced NF-kappaB signaling and promoted apoptosis	Provides mechanistic target-validation evidence only; corresponding therapeutic studies are lacking.	[107]
	Colorectal cancer	HTRA1 overexpression or knockdown	HTRA1 stabilizes LATS2 and restrains Hippo/YAP1-driven EMT and anoikis resistance		[73]
	Pancreatic cancer	HTRA1 overexpression or knockdown	Notch-1-associated inhibition of growth and migration		[79]
	Colorectal cancer	HTRA1 overexpression or knockdown	Interaction with SLC7A11 through the Kazal domain, suppressing ferroptosis and promoting drug resistance		[98]
	Ovarian cancer	Purified wild-type or catalytically inactive HTRA1; HTRA1 overexpression or knockdown	Protease-dependent XIAP degradation and cisplatin sensitization		[93]
	Ovarian cancer	Protease-inactive HTRA1 mutant	Protease activity-dependent suppression of EGFR/AKT signaling and anoikis resistance		[84]

PDAC: pancreatic ductal adenocarcinoma.

## Data Availability

No new data were created or analyzed in this study. Data sharing is not applicable to this article.

## Abbreviations

The following abbreviations are used in this manuscript:

HTRA1	High-temperature requirement A serine peptidase 1
ECM	Extracellular matrix
PD	Protease domain
TGF-β	Transforming growth factor-β
MMP	Matrix metalloproteinases
ADAMTS	A Disintegrin and Metalloproteinase with Thrombospondin motifs
CARASIL	Cerebral autosomal recessive arteriopathy with subcortical infarcts and leukoencephalopathy
AMD	Age-related macular degeneration
AD	Alzheimer’s disease
MSCs	Mesenchymal stem cells
KFB	Keloid fibroblasts
DCM	Dilated cardiomyopathy
TGFBR2	Transforming growth factor beta receptor II
BMP	Bone morphogenetic protein
RA	Rheumatoid arthritis
LPS	Lipopolysaccharide
OA	Osteoarthritis
IVDD	Intervertebral disc degeneration
TME	Tumor microenvironment
EMT	Epithelial–Mesenchymal transition
YAP	Yes-associated protein
TAZ	Transcriptional coactivator with PDZ-binding motif
LATS2	Large tumor suppressor kinase 2
α-SMA	alpha-smooth muscle actin
XIAP	X-linked inhibitor of apoptosis protein
PDAC	pancreatic ductal adenocarcinoma
LNP	lipid nanoparticle
CRC	colorectal cancer
